# Anticipation and Motivation as Predictors of Leisure and Social Enjoyment and Engagement in Young People With Depression Symptoms: Ecological Momentary Assessment Study

**DOI:** 10.2196/74427

**Published:** 2025-08-13

**Authors:** Angad Sahni, Ciara McCabe

**Affiliations:** 1 Department of Psychology, School of Psychology and Clinical Language Sciences, University of Reading, Whiteknights Campus, Reading, RG6 6AL, United Kingdom, 44 (0) 118 378 5450, 44 (0) 118-378-8523

**Keywords:** depression, internet, app, EMA, reward processing, ecological momentary assessment, leisure, social, activity, engagement, enjoyment, anticipation, motivation., mobile phone

## Abstract

**Background:**

Participating in leisure and social activities can alleviate depression symptoms, yet effective strategies to enhance enjoyment and maintain long-term engagement remain scarce. Gaining insight into the reward subcomponents that influence daily experiences and drive behavior could uncover novel targets for intervention.

**Objective:**

This study examines the role of anticipation and motivation in predicting enjoyment and engagement in leisure activities and socializing among young people, and how these relationships are moderated by depression severity, using intensive longitudinal ecological momentary assessments.

**Methods:**

Participants (N=80; mean age 20, SD 2.3 years) used the Psymate2 smartphone app to report mood, enjoyment, current and anticipated activities, and social company 7 times daily for 6 days. Activity categories were relaxation, exercise, other leisure, work or school, studying, chores, shopping, hygiene, eating or drinking, and traveling, and company categories were partner, friends, family, colleagues, acquaintances, strangers, and nobody. Anticipation (anticipatory pleasure and expectation) and motivation (interest and preference) for upcoming activities were rated on Likert scales. Participants were grouped by depression severity, measured using the Mood and Feelings Questionnaire (MFQ): high (HD, MFQ ≥27, N=42), moderate (MD, MFQ 16‐27, N=16), and low, that is, controls (C, MFQ ≤16, N=22). Totally, 2316 assessments met inclusion criteria.

**Results:**

Leisure activities (relaxation, exercise, and other leisure) and social company (partner, friends, and family) were rated most enjoyable across all groups. Higher depression symptoms were associated with reduced enjoyment of studying (β=−.03; *P*=.005), eating or drinking (β=−.02; *P*=.02), and other leisure activities (β=−.02; *P*=.02), as well as lower engagement in work or school (β=−.26; *P*=.02) and hygiene (β=−.08; *P*=.03), and increased inactivity (β=.17; *P*=.03). Time-lagged multilevel analyses showed that anticipatory pleasure predicted greater enjoyment across all activities (β=.12; *P*<.001) and social contexts (β=.33; *P*<.001), with consistent effects in controls and the high depression group. However, the more an activity was expected to happen, the less enjoyment was experienced in the whole sample (β=−.006; *P*=.001) and high depression group (β=−.008; *P*=.001) but not controls. Anticipatory pleasure and motivation (preference) predicted leisure engagement in the whole sample (β=.19, *P*=.003; β=.11, *P*<.001) and controls (β=.43, *P*=.005; β=.17, *P*=.048) but not the depression groups. Anticipatory pleasure predicted only leisure engagement in the high depression group when predictors and outcomes were matched for the same event (β=.22; *P*=.001). Anticipatory pleasure predicted social engagement in the whole sample (β=.095; *P*=.047) and controls (β=.34; *P*=.003), but not in the depression groups.

**Conclusions:**

These findings highlight the importance of anticipatory pleasure and intrinsic motivation in shaping young people’s engagement and enjoyment of daily activities. Structured or externally driven contexts may dampen enjoyment—especially among those with depression—underscoring the need for novel interventions targeting anticipation and motivation to enhance sustained participation in rewarding activities, leading to improved well-being in individuals with depression.

## Introduction

Major depressive disorder is a leading cause of disease burden among young people worldwide, yet current treatments offer only moderate effectiveness [1,2]. A key challenge in depression treatment is that individuals with depression engage less in rewarding activities, likely due to anhedonia—a diminished ability to anticipate and experience pleasure [3-5]. Furthermore, depression also encompasses deficits in the motivation to engage in rewarding experiences [6]. These deficits in reward function could lead to inactivity, which is particularly problematic as reduced engagement in physical and social activities perpetuates symptoms, reinforcing a cycle of low mood and withdrawal. Additionally, loneliness not only increases depression risk [7,8] but also predicts future antidepressant use [9] underscoring the urgent need for interventions that promote sustained participation in mood-boosting activities [10-14].

A growing body of research highlights the protective role of leisure activities—those that are inherently enjoyable and recreational—in reducing depression rates in the general population [15-19] and in patients [20,21], compared with functional activities such as work or chores. Similarly, social interactions, particularly with close friends and family, appear to be more effective in alleviating depression symptoms than solitary activities or interactions with acquaintances [22]. These findings support the rationale for interventions such as behavioral activation, which encourages individuals to increase engagement in pleasurable activities to enhance positive reinforcement [21,23]. However, despite its effectiveness, behavioral activation does not outperform other treatments, likely due to an incomplete understanding of the reward mechanisms that drive sustained engagement [24,25].

To optimize interventions, a more nuanced understanding of reward function is needed. Reward processing involves distinct subcomponents, including anticipation, motivation, and enjoyment, which collectively influence approach behavior [26,27]. According to the Temporal Experience of Pleasure model, anticipation plays a crucial role in shaping motivation, which then drives behavioral engagement [28,29].

Reward dysfunction is well documented in depression (Ma X, Sahni A, McCabe C, unpublished data, 2024) [30,31]. For example, higher depression symptoms in adolescents and college students are shown to be associated with lower social pleasure using the Domains of Pleasure Scale [32], and lower consummatory pleasure using the Snaith-Hamilton Pleasure Scale [33] and the Fawcett-Clark Pleasure Capacity Scale [34]. We have also found lower anticipatory and consummatory pleasure in young people with depression using the Temporal Experience of Pleasure Scale [35,36]. Furthermore, studies find that depressed adolescents have reduced pleasure and motivation for hobbies, food or drinks, social activities, and sensory experiences using the Dimensional Anhedonia Rating Scale [37]. The recently developed Positive Valence System Scale [38] incorporates nearly all the reward subprocesses, such as anticipatory pleasure, consummatory pleasure, and motivated behavior and effort, and has shown that deficits in all subprocesses correlate with depressive symptoms in a college student sample [38].

Laboratory-based tasks have found lower motivation to exert effort for rewards with increasing depression symptoms in young people [36]. Similarly, studies find that anhedonia is associated with a lower frequency of high-effort choices in a young community sample [39-41] and in young people with subthreshold depression [42]. Taken together, there is evidence for reward subcomponent-processing deficits in young people that could underpin their depression symptoms. However, surveys and experimental tasks still fail to capture the dynamic real-world nature of reward-driven behavior (Ma X, Sahni A, McCabe C, unpublished data, 2024) [30,43], leaving critical gaps in our understanding of how anticipation and motivation influence engagement in daily activities in real time.

Ecological momentary assessment (EMA) offers a powerful solution to this limitation by tracking real-time experiences within natural environments using smartphone-based assessments [44-47]. EMA studies have demonstrated that social and physical activities protect against depression [48] and that individuals with depression experience fewer positive daily events and social interactions, predicting lower well-being even a decade later [49]. Critically, while one study has shown that anticipatory pleasure predicts subsequent enjoyment of an activity [50] and another found that depressive symptoms weaken the link between anticipation and behavior [51], these findings remain incomplete. Existing EMA studies have yet to examine motivation as a predictor of activity engagement, despite its central role in approach behavior. Furthermore, it is unknown whether anticipation and motivation specifically predict engagement in key mood-boosting activities such as leisure and socializing—activities known to be crucial for mental health [52,53].

To address these gaps, this study investigates how anticipation and motivation influence enjoyment and engagement in leisure activities and social contexts using EMA. Adopting a methodological approach similar to the work on schizophrenia by Edwards et al [54], we hypothesize that individuals with higher depressive symptoms will exhibit weaker associations between anticipation, motivation, and subsequent engagement. By providing real-world evidence on the role of reward subcomponents in shaping behavior, this study could identify novel targets for reward-based interventions aimed at improving mental health outcomes.

## Methods

### Power Analysis

We conducted a priori power analysis using PowerAnalysisIL designed for EMA [55] using data provided by Li et al [50] (Table S1 and Figure S2 in Multimedia Appendix 1). Analysis suggested that 80% power could be achieved with a sample size of 50, consistent with estimates from multilevel logistic [56] and linear models [57] using our design.

### Recruitment

Young people (N=95), aged 16‐25 years with varying depression symptoms, were recruited from local schools and universities.

### Patient and Public Involvement and Piloting

Based on feedback from piloting sessions with young people, we revised the activity categories and allocated time for app troubleshooting in our study briefing sessions. We also assessed completion time for questionnaires and participant feedback. We found the daily burden manageable, supporting our decision to retain the questionnaire structure.

### Baseline Demographics

Participants completed the Mood and Feelings Questionnaire (MFQ; ≥27 cut off for clinical depression) [58]. Higher scores indicate higher depression symptoms. The 33-item questionnaire has been shown to have excellent internal reliability in adolescents (Cronbach α=0.91-0.93) [59]. It is a widely used and validated questionnaire to examine depressive symptoms in young people.

### EMA Assessments

Similar to an EMA study in schizophrenia [54] at each assessment (beep), participants rated their mood (negative affect and positive affect) and then selected their current activity from categories and rated their enjoyment. They then selected current company from a selection of categories and rated their enjoyment. Next, they selected their future activity from the same categories and rated their anticipatory pleasure, expectation (likelihood of activity happening), and motivation (interest and preference). They then selected future company from categories and rated their anticipatory pleasure. The motivation questions were posed indirectly so as not to intervene or influence, and this style of questioning limits the potential for the participants to change their behavior in response to the question [54] (Table 1).

**Table 1.** Description of the ecological momentary assessment questionnaires, showing the questions used to capture the reward subprocesses and categories of activities and company, and how they were rated.

Reward subprocess	Question	Categories and ratings
Mood	“Right now I feel…”	Cheerful, ashamed, annoyed, enthusiastic, relaxed, anxious, satisfied, lonely, insecure, down, guilty. 1 = Not at all to 7 = Very much
Current physical	What were you doing just before the beep went off? (*Select one*)	Relaxing, work or school, studying, chores, shopping, hygiene, eating or drinking, traveling, social media, exercising, other leisure activity, nothing.
Enjoyment	How much are you enjoying this activity?	1 = Not at all to 7 = Very much
Current company	Who are you with? (Select one)	Partner, friends, family, colleagues, acquaintances, strangers, nobody.
Enjoyment	How much are you enjoying being in this company?	1 = Not at all to 7 = Very much
Future physical	What activity will you be doing after this one? (Select one)	Relaxing, work or school, studying, chores, shopping, hygiene, eating or drinking, traveling, social media, exercising, other leisure activity, nothing.
Anticipatory pleasure	How much do you think you will enjoy this activity?	1 = Not at all to 7 = Very much
Expectation	What do you think are the chances this activity will occur?	Rated from 0% to 100%.
Motivation (interest)	How interested are you in this activity?	1 = Not at all to 7 = Very much
Motivation (preference)	Would you prefer to do something else?	1 = Not at all to 7 = Very much
Future company	Who will you be with? (Select one)	Partner, friends, family, colleagues, acquaintances, strangers, nobody.
Anticipatory pleasure	How much do you think you will enjoy this company?	1 not at all to 7 = Very much

### Procedure

The experimenter met with each participant to brief them on the app. Participants were then required to log on and fill in their age, gender, ethnicity, and the MFQ. EMA assessments began the next day. Participants were asked to respond to each assessment as soon as possible; otherwise, they would expire.

We collected data 7 times a day, between 8:30 AM and 10 PM, for 6 days, during the period July 2022 to October 2023. There was at least a 45-minute delay between each semirandom assessment, which took approximately 1 minute to complete and expired after 20 minutes. This sampling frequency and questionnaire design has been shown to encourage compliance and reduce burden in young people and those with mood disorders [56,57,60,61]. We contacted participants on days 2 and 5 to check that they were receiving notifications and to troubleshoot any problems.

At the end of the study, we collected app user experiences (Table S2 in Multimedia Appendix 1), and participants were debriefed and advised to contact their general practitioner or the mental health charity, the Samaritans, if they were concerned about their mood.

### Data Analysis

Fisher exact tests, ANOVAs, and chi-square statistics were used to examine group differences in the demographic data. We prepared the EMA data using *esmpack* in R (version 4.4.1; R Core Team, 2024) [62]. Activity and company enjoyment and engagement were calculated across participants and ranked from highest to lowest.

To check for potential confounds, we ran multilevel regression models using the lme4 package in R with mood (negative affect and positive affect), assessment time (1-7), and assessment day (1-6) as predictors of anticipation, motivation, and enjoyment. Predictors were subject-mean centered and represented at level 1 and symptoms were level 2 fixed effects. All multilevel models were represented by a subject-level random intercept. As assessment time and mood covaried, they were added to all multilevel analyses as covariates (Table S3 in Multimedia Appendix 1).

We next ran multilevel models with depression as a continuous variable (MFQ) as a predictor of anticipation, motivation, and enjoyment across all activity, then specifically leisure (relaxing, other leisure, and exercise) and functional activity (work or school, studying, chores, shopping, hygiene, eating or drinking, and traveling). We also ran multilevel models with depression as a continuous variable as a predictor of anticipatory pleasure and enjoyment across all company and then specifically social (partner, friends, and family) and nonsocial company (nobody). We then examined the effects of depression symptoms on enjoyment and engagement for each specific activity and specific company, using separate standard linear regression models.

The Temporal Experience of Pleasure cycle shows that anticipation and motivation are temporally predictive of enjoyment and engagement in activities [28,29]. This is the basis of our theoretical framework (Figure S1 in Multimedia Appendix 1) for examining the temporal dynamics using time-lagged analyses. Using anticipation and motivation as predictors at (*t*−1), we modeled activity enjoyment and engagement (0 = functional, 1 = leisure) as outcomes at (*t*) using multilevel linear and logistic models, respectively. Similarly, using anticipation at (*t*−1), we modeled company enjoyment and engagement (0 = nonsocial, 1 = social) as outcomes at (*t*). We reported data for the whole sample (WS) and participants by depression symptom severity; controls (C, score of ≤16 on MFQ), moderate (MD, score of >16 and <27 on MFQ), and high depression symptoms (HD, score of ≥27 on MFQ). Splitting the sample into groups, similar to Li et al [50], allows us to compare the 4 main predictors (anticipatory pleasure, interest, preference, and expectation) in the same model while controlling for each other, across each of the 4 groups.

As participants may anticipate doing something but then do something else, we also examined the data when the predictors (anticipation and motivation) and outcomes (enjoyment and engagement) were matched for the same events, for example, anticipatory pleasure for exercise predicting enjoyment of exercise. However, matched data can mean much fewer assessments and possible model overparameterization.

### Ethical Considerations

The study adhered to ethical standards (Revised Declaration of Helsinki 2008) and was approved by the University of Reading Psychology Department ethics committee (REC number 2021‐120-CM). All participants provided written informed consent after reviewing the information sheets. To preserve participant privacy, no identifiable features were included in the manuscript and supplementary materials. After completion, participants were compensated by SONA credits [63].

## Results

### Overview

A total of 2426 questionnaires were collected from 95 participants. We excluded participants who had <30% threshold of assessments (N=15) [64,65], and the mean compliance rate was 69%, similar to previous EMA studies [66]. About 1.5% of assessments were incomplete and therefore removed from analysis.

### Demographics

The final sample consisted of 2316 questionnaires. Participants self-identified as White (63/80, 78.8%), Black (4/80, 5%), Asian (9/80, 11%), Mixed (2/80, 2.5%), and Other (2/80, 2.5%). The depression groups differed on symptoms as expected and also for age and assessment period but not for gender, ethnicity, compliance, or assessment delay (Table 2).

**Table 2.** Description of demographics of the whole sample and the 3 groups: controls, moderate depression, and high depression groups^a^.

Characteristics	Whole sample (n=80, 2316)	C^b^ (n=22, 610)	MD^c^ (n=16, 439)	HD^d^ (n=42, 1267)	Statistic	*P* value
Age (years)	20.12 (2.3)	21.55 (2.79)	19.81 (2.04)	19.5 (1.77)	*F*_2,77_=6.77	.002
Gender (% male)	27.5	27.3	31.2	26.2		.971^e^
Gender split (female/male/other)	57/22/1	16/6/0	11/5/0	30/11/1		
Compliance, n (%)	68.9 (16.43)	66 (15.61)	65.3 (18.21)	71.8 (15.99)	*F*_2,77_=1.39	.255
Assessment delay (minutes)	118 (41.05)	120 (42.67)	116 (40.83)	118 (40.38)	*F*_2,1559_=0.68	.508
Assessment period^f^						
Holiday	879	370	197	312	*χ*^2^_2_=238.01	<.001
Term time	1437	240	242	955		
Ethnicity, n (%)						
White	63 (78.8)	19 (86.3)	13 (81.3)	31 (73.8)		.816^e^
Black	4 (5)	0 (0)	1 (6.3)	3 (7.1)		
Asian	9 (11)	2 (9.1)	1 (6.3)	6 (14.3)		
Mixed	2 (2.5)	0 (0)	1 (6.3)	1 (2.4)		
Other	2 (2.5)	1 (4.5)	0 (0)	1 (2.4)		
MFQ^g^	25.64 (13.1)	8.86 (3.67)	21 (3.04)	36.2 (6.83)	*F*=185.34 (2,77)	<.001
Medication for depression	8/80	0	0	8	N/A^h^	N/A

a N=sample size, no of assessments. Values are mean (SD) unless stated otherwise. Holiday dates: <18 years = July 21 to September 6, 2022, and July 21 to September 26, 2022; >18 years = December 19, 2022, to January 3, 2023. Any other dates =Term time.

b C: controls.

c MD: moderate.

d HD: high.

e Fisher exact test (2-tailed).

f Values are the number of assessments in holiday and term time.

g MFQ: Mood and Feelings Questionnaire.

h Not applicable.

### What Activity Do Young People Enjoy and Engage With?

The leisure activities “exercising,” “relaxing,” and “other leisure” were rated most enjoyable (Table S4 in Multimedia Appendix 1). Young people engaged most frequently in “relaxing” and “other leisure” activities, followed by “eating or drinking” and then “work or school” and “studying” (Multimedia Appendix 2).

### What Company Do Young People Enjoy and Engage With?

Being with “friends,” “family,” and “partner” was rated highest for enjoyment (Table S5 in Multimedia Appendix 1). Although young people spent more time alone than with others, they were mostly with “friends,” “family,” and “partner” when socializing (Multimedia Appendix 2).

### Dimensional Analyses

#### Are Depression Symptoms Associated With Reduced Anticipation, Motivation, and Enjoyment of Activity?

As depression increased, anticipation (anticipatory pleasure and expectation), motivation (interest and preference), and enjoyment decreased across all activities (Table S6 in Multimedia Appendix 1). When examining data for leisure and functional activity, enjoyment and expectation also decreased as depression increased (Table S7 in Multimedia Appendix 1).

#### Are Depression Symptoms Associated With Anticipation and Enjoyment of Company?

As depression increased, anticipatory pleasure and enjoyment decreased across all company (Table S8 in Multimedia Appendix 1). Anticipatory pleasure and enjoyment of social company (friends, family, and partner) and enjoyment of non–social company (nobody) decreased as depression increased (Table S9 in Multimedia Appendix 1).

#### Are Depression Symptoms Associated With Enjoyment and Engagement in Specific Activities and Specific Company?

As depression increased, enjoyment for “studying,” “eating or drinking,” and “other leisure” activities decreased (Table S10 in Multimedia Appendix 1), and as depression increased, young people reported doing more “nothing,” less “work or school,” and “hygiene” (Table S11 in Multimedia Appendix 1). Depression did not predict enjoyment or engagement with any specific company.

### Categorical Analyses

#### Time-Lagged Analyses

Using multilevel time-lagged linear regressions, anticipatory pleasure positively predicted enjoyment across all activity categories in the WS, controls, and in those with high depression symptoms (HD) (Table 3). Expectation negatively predicted enjoyment across all activity categories in WS and HD (Table 3).

##### Predicting Activity Enjoyment and Company Enjoyment

Anticipatory pleasure positively predicted enjoyment across all company categories in all groups (Table 4). Results were similar when predictors and outcomes were matched for the same events (Table S12 in Multimedia Appendix 1).

**Table 3.** Reward predictors of activity enjoyment^a^.

	Whole sample (n=1514)			C^b^ (n=389)			MD^c^ (n=275)			HD^d^ (n=850)		
Predictors (*t*−1)	β	95% CI	*P* value	β	95% CI	*P* value	β	95% CI	*P* value	β	95% CI	*P* value
Intercept	4.426	4.189 to 4.663	<.001	4.677	4.291 to 5.063	<.001	4.313	3.741 to 4.885	<.001	4.290	3.965 to 4.615	<.001
Anticipatory pleasure	.118	0.051 to 0.185	<.001	.160	0.019 to 0.301	.03	.082	−0.089 to 0.253	.34	.118	0.034 to 0.202	.006
Expectation	−.006	−0.01 to −0.002	.001	−.008	−0.016 to 0	.08	.000	−0.008 to 0.008	.99	−.008	−0.012 to −0.004	.001
Motivation (interest)	.057	−0.006 to 0.12	.08	.000	−0.137 to 0.137	.99	.104	−0.051 to 0.259	.19	.051	−0.029 to 0.131	.22
Motivation (prefer)	.027	−0.016 to 0.07	.21	.016	−0.062 to 0.094	.70	−.034	−0.138 to 0.07	.52	.056	−0.003 to 0.115	.058

a Time-lagged predictors of enjoyment of activities in the whole sample, controls, moderate depression, and high depression groups. Time-lagged linear regressions, n = number of assessments. Controlled for assessment time and mood (negative affect and positive affect). When age and assessment period were added as extra covariates, results remained the same.

b C: controls.

c MD: moderate.

d HD: high.

**Table 4.** Reward predictors of company enjoyment.^a^

	Whole sample (n=1516)			C^b^ (n=389)			MD^c^ (n=277)			HD^d^ (n=850)		
Predictors (*t*−1)	β	95% CI	*P* value	β	95% CI	*P* value	β	95% CI	*P* value	β	95% CI	*P* value
Intercept	5.230	5.038-5.422	<.001	5.585	5.23-5.94	<.001	5.482	5.055-5.909	<.001	4.952	4.705-5.199	<.001
Anticipatory pleasure	.329	0.278-0.38	<.001	.342	0.246-0.438	<.001	.244	0.119-0.369	<.001	.341	0.272-0.41	<.001

a Time-lagged predictors of enjoyment of company in the whole sample, controls, moderate depression, and high depression groups. Time-lagged linear regressions, n = number of assessments. Controlled for assessment time and mood (negative affect and positive affect). When age and assessment period were added as extra covariates, results remained the same.

b C: controls.

c MD: moderate.

d HD: high.

##### Predicting Leisure Enjoyment and Social Company Enjoyment

When looking specifically at leisure (exercising, relaxing, and other leisure activity), we found that motivation (interest) positively predicted leisure enjoyment at trend in the WS (*P*=.06) (Table S13A in Multimedia Appendix 1). Anticipatory pleasure positively predicted enjoyment of social company (friends, family, and partner) in both the WS and HD groups and at trend in the MD group (*P*=.055) (Table S13B in Multimedia Appendix 1).

##### Predicting Leisure Engagement and Social Company Engagement

Using multilevel time-lagged logistic regressions, anticipatory pleasure and motivation (preference) positively predicted engagement in leisure in the WS and controls (Table 5). When predictors and outcomes were matched for the same leisure events, anticipatory pleasure positively predicted engagement in leisure activities in the WS and HD group (Table S14A in Multimedia Appendix 1). Expectation negatively predicted engagement in leisure in the WS, controls, and HD groups (Table 5).

Anticipatory pleasure positively predicted engagement in social company in the WS and controls and in the matched condition (when predictors and outcomes were for the same social company) (Table 6 and S14B in Multimedia Appendix 1). Anticipatory pleasure did not predict engagement in social company in the MD or HD groups.

**Table 5.** Reward predictors of leisure activity engagement (leisure vs functional activity engagement)^a^.

	Whole sample (n=1383)			C^b^ (n=365)			MD^c^ (n=257)			HD^d^ (n=761)		
Predictors (*t*−1)	β	95% CI	*P* value	β	95% CI	*P* value	β	95% CI	*P* value	β	95% CI	*P* value
Intercept	−.633	−0.943 to −0.323	<.001	−.514	−1.161 to 0.133	.12	−.973	−1.688 to −0.258	.008	−.620	−1.032 to −0.208	.003
Anticipatory pleasure	.194	0.069 to 0.319	.003	.426	0.13 to 0.722	.005	.232	−0.105 to 0.569	.18	.129	−0.028 to 0.286	.10
Expectation	−.014	−0.022 to −0.006	<.001	−.032	−0.05 to −0.014	.001	−.005	−0.021 to 0.011	.55	−.013	−0.021 to −0.005	.003
Motivation (interest)	−.056	−0.176 to 0.064	.35	−.142	−0.422 to 0.138	.32	.006	−0.29 to 0.302	.97	−.079	−0.228 to 0.07	.30
Motivation (prefer)	.105	0.023 to 0.187	.011	.165	0.002 to 0.328	.048	.090	−0.116 to 0.296	.39	.087	−0.023 to 0.197	.12

a Time-lagged predictors of engagement in leisure activities in the whole sample, controls, moderate depression, and high depression groups. Time-lagged logistic regressions, n = number of assessments. Leisure versus functional activity outcomes were coded as 1 and 0, respectively. Controlled for assessment time and mood (negative affect and positive affect). When age and assessment period were added as extra covariates, results remained the same.

b C: controls.

c MD: moderate.

d HD: high.

**Table 6.** Reward predictors of social company engagement (social vs non–social company engagement)^a^.

	Whole sample (n=1413)			C^b^ (n=361)			MD^c^ (n=270)			HD^d^ (n=782)		
Predictors (*t*−1)	β	95% CI	*P* value	β	95% CI	*P* value	β	95% CI	*P* value	β	95% CI	*P* value
Intercept	−.046	−0.279 to 0.187	.703	−.056	−0.556 to 0.444	.825	−.024	−0.406 to 0.358	.902	−.053	−0.392 to 0.286	.760
Anticipatory pleasure	.095	0.001 to 0.189	.047	.343	0.116 to 0.57	.003	.068	−0.177 to 0.313	.583	.033	−0.083 to 0.149	.570

a Time-lagged predictors of engagement in social company in the whole sample, controls, moderate depression, and high depression groups. Time-lagged logistic regressions, n = number of assessments. Social versus nonsocial activity outcomes were coded as 1 and 0, respectively. Controlled for assessment time and mood (negative affect and positive affect). When age and assessment period were added as extra covariates, results remained the same.

b C: controls.

c MD: moderate.

d HD: high.

## Discussion

### Principal Results

To our knowledge, this is the first study to demonstrate in real-world settings that anticipatory pleasure reliably predicts future enjoyment across various physical activities and social contexts in young people. Anticipatory pleasure and motivation (preference) predicted leisure engagement, and anticipatory pleasure predicted social engagement in the WS and controls but not in individuals with depression symptoms. However, when measured for the same specific event, anticipatory pleasure does predict leisure engagement in those with higher depression symptoms, indicating a potential target for intervention.

### Comparison With Prior Work

While prior research has established that depression blunts reward-related processes (Ma X, Sahni A, McCabe C, unpublished data, 2024), most studies rely on retrospective reports or laboratory tasks that fail to capture the temporal dynamics of reward processing in daily life. By leveraging EMA, this study provides novel insights into how anticipation and motivation dynamically shape behavior in real-world contexts. Consistent with prior work [50,67], we found that individuals with higher depression symptoms reported less enjoyment across various activities. However, our study extends these findings by showing that depression is also linked to a broader dampening of anticipation and motivation across functional, leisure, and social experiences. This supports the idea that depression is associated not only with reduced momentary pleasure but also with a diminished ability to anticipate and seek out rewarding experiences.

Our study also provides a nuanced perspective on real-world activity deficits in depression. Individuals with higher depression symptoms reported lower enjoyment in activities such as studying, eating or drinking, and other leisure activities, as well as reduced engagement in work or school and hygiene. Importantly, depression was associated with doing more “nothing,” a behavioral pattern consistent with wearable-monitor studies showing reduced activity in depression [68,69]. However, our results go beyond previous findings by contextualizing these activity deficits within specific daily-life behaviors. The reduction in work or school and hygiene aligns with known risk factors for depression, such as academic underachievement [70] and self-neglect [71]. These findings suggest that ecological momentary interventions (EMIs) could be developed to support goal setting, progress tracking, and real-time encouragement for maintaining daily routines [47,72].

Crucially*,* we demonstrate for the first time that anticipatory pleasure significantly predicts future enjoyment across all physical activities and social contexts. Anticipatory pleasure and motivation (preference) predicted leisure engagement, and anticipatory pleasure predicted social engagement in the WS and controls but not in individuals with depression symptoms. However, anticipatory pleasure did predict leisure engagement in those with high depression symptoms, when assessed for the same specific event. This suggests that while individuals with depression may struggle to generalize anticipation across contexts, if they can be encouraged to anticipate specific leisure activities, they may be more likely to engage in them, which could strengthen the anticipation-engagement link over time.

Our findings support the development of interventions that could exploit the time-lagged relationships such as episodic future thinking (EFT) [73], a cognitive exercise that uses mental imagery to anticipate the feelings associated with future positive activities. Preliminary data suggest that EFT can increase anticipatory pleasure using both traditional scales [74-76] and EMA [77] in healthy controls and those with major depressive disorder. Therefore, we propose that an EFT-based EMI could be developed and tested to see whether it could increase engagement in leisure and social activities in young people with depression symptoms.

A striking finding was that higher expectation (likelihood of activity happening) was associated with lower enjoyment, especially in those with high depression symptoms. This aligns with our finding that the least enjoyable activities were work or school and studying, activities that are usually scheduled and therefore highly expected. Consistent with this, we found that enjoyment for activities such as studying decreased as depression increased and that higher expectation predicted more engagement with functional versus leisure activities across groups. These findings are supported by previous reports that show that activity scheduling reduces enjoyment, potentially due to diminished spontaneity [78].

Our findings could be explained by Self-Determination Theory [79], whereby activities that are externally imposed (such as school or studying) and extrinsically motivated (to pass examinations) tend to be less enjoyable, especially if autonomy is low, contrasted with activities that are intrinsically motivated such as leisure (inherently enjoyable). In depressed individuals, the lack of intrinsic reward and reduced agency may amplify the negative experience of expected, scheduled tasks. As the least enjoyable activities in this study are the ones most likely scheduled—this aligns with the Self-Determination Theory that autonomy is key to enjoyment and well-being. Moreover, as individuals with depression tend to hold negative core beliefs and cognitive distortions [80], negative biases could lead to negative perceptions of predictable activities, such as school or work, sustaining low enjoyment.

Taken together, highly expected activities are often externally imposed, reducing autonomy and intrinsic motivation. For youth with depression, these activities—especially when perceived as unpleasant—could confirm negative schemas about being powerless or trapped [80], which could lead to lower enjoyment and possibly deeper anhedonia. Future research should explore whether EMIs that promote spontaneous enjoyable activities, while addressing negative cognitive biases, could enhance activity engagement in depression.

### Limitations

While our study offers significant contributions, several limitations must be acknowledged. First, although EMA captures real-time experiences, self-report measures remain subject to bias. Future studies could integrate passive-sensing data (eg, activity trackers and voice recordings) to objectively assess engagement [81]. Second, our sample consisted of young adults, limiting generalizability to older populations. In future studies, time-lagged relationships should be investigated in a broader demographic. Third, while we demonstrated the predictive role of anticipation and motivation, causality remains uncertain. Experimental studies using reward-training interventions could further elucidate these mechanisms.

### Conclusions

Our results support anticipation and intrinsic motivation as novel intervention targets for real-time, technology-driven approaches to managing depression. By leveraging digital tools to enhance anticipation and motivation, future interventions could empower individuals to engage with rewarding experiences, ultimately improving mental health outcomes.

## Supplementary material

10.2196/74427 Multimedia Appendix 1 Theoretical framework, power analysis, symptoms association with enjoyment and engagement, and time-lagged analysis for the same events.

10.2196/74427 Multimedia Appendix 2 Activities and social company that young people engaged in.

## Abbreviations

C: Controls
EFT: episodic future thinking
EMA: ecological momentary assessment
EMI: ecological momentary intervention
HD: High depression
MD: Moderate depression
MFQ: Mood and Feelings Questionnaire
WS: whole sample
