# Supplementary material for: Anticipation and Motivation as Predictors of Leisure and Social Enjoyment and Engagement in Young People With Depression Symptoms: Ecological Momentary Assessment Study
Source: JMIR Ment Health. 2025 Aug 13;12:e74427. doi: 10.2196/74427 (PMC12349889; doi:10.2196/74427)
Supplement: Multimedia Appendix 1 [file mental-v12-e74427-s001.docx]

**Supplementary References**

This supplemental material has been provided by the authors to give readers additional information.

**Figure S1. Visual Model of the Theoretical Framework.**

Adapted from the Temporal Experience of Pleasure (TEP) cycle by Kring and Barch [1], the visual model of the theoretical framework of the time-lagged models is shown in Figure S1. The models examine how anticipation (anticipatory pleasure, expectation) and motivation (interest, preference) (at t-1) predict enjoyment and engagement (at t) in leisure and social activities.

The TEP cycle demonstrates that, after experiencing enjoyment, the subjective pleasure associated with an activity is updated. The next time the same activity is anticipated, the updated subjective pleasure is reflected in its anticipatory pleasure.

*Anticipation (t-1)*

*Engagement (t)*

*Updating/Activating*

*Activity-Related Pleasure*

Anticipatory Pleasure

Expectation

**Figure S1.** The theoretical framework of the time-lagged models, adapted from the temporal experience of pleasure (TEP) cycle by Kring and Barch [1].

*Motivation (t-1)*

Interest

Preference

*Enjoyment (t)*

**Power analysis**

Power calculations for EMA studies are complicated due to the intensive longitudinal study designs and are thus rarely reported. Using a recently developed app specifically designed for EMA power analysis PowerAnalysisIL [2] we conducted an *a priori* power analysis. We requested data from Li et al., as they also examined the relationship between depression symptoms and reward processes in an EMA study in young people (N=100) [3]. We utilised Model 2 in the app where Level 2 is represented by a subject-level random intercept and the Level 2 predictor (depression score) predicts anticipatory pleasure [2]. At 1000 Monte Carlo replicates, the relevant parameters (Table S1) extracted from their data created a power curve for the effect of the Level 2 variable (effect of interest in our study in Table S8 – depression predicting EMA measures). This analysis suggested that 80% power could be achieved with a sample size of 50 (Figure S2). Consistent with this estimates from multilevel logistic [4] and linear models [5] also suggest a minimum sample size of 50 for our design.

**Table S1.** Parameters extracted from Li et al. study data.

| **Table S1** |  |  |
| --- | --- | --- |
| **Parameters** | **Notation** | **Model Estimate** |
| Time points |  | 44 (max = 56); 79% mean compliance. |
| Mean of Level 2 continuous variable (BDI Total) | *μ_W_* | 16.08 |
| Std. Dev. of BDI Total | *σ_W_* | 9.58 |
| Fixed Intercept | *β_00_* | 7.31 |
| Effect of BDI Total (level 2) on Level 1 Intercept | *β_01_* | -0.049 |
| Std. Dev. of Level 1 error (residuals) | *σ_ε_* | 1.60 |
| Autocorrelation of Level 1 error | *ρ_ε_* | 0.15 |
| Std. Dev. of Random Intercept (subject level) | *σ_v0_* | 1.09 |

**Figure S2.** Power curves generated in the app, PowerAnalysisIL.


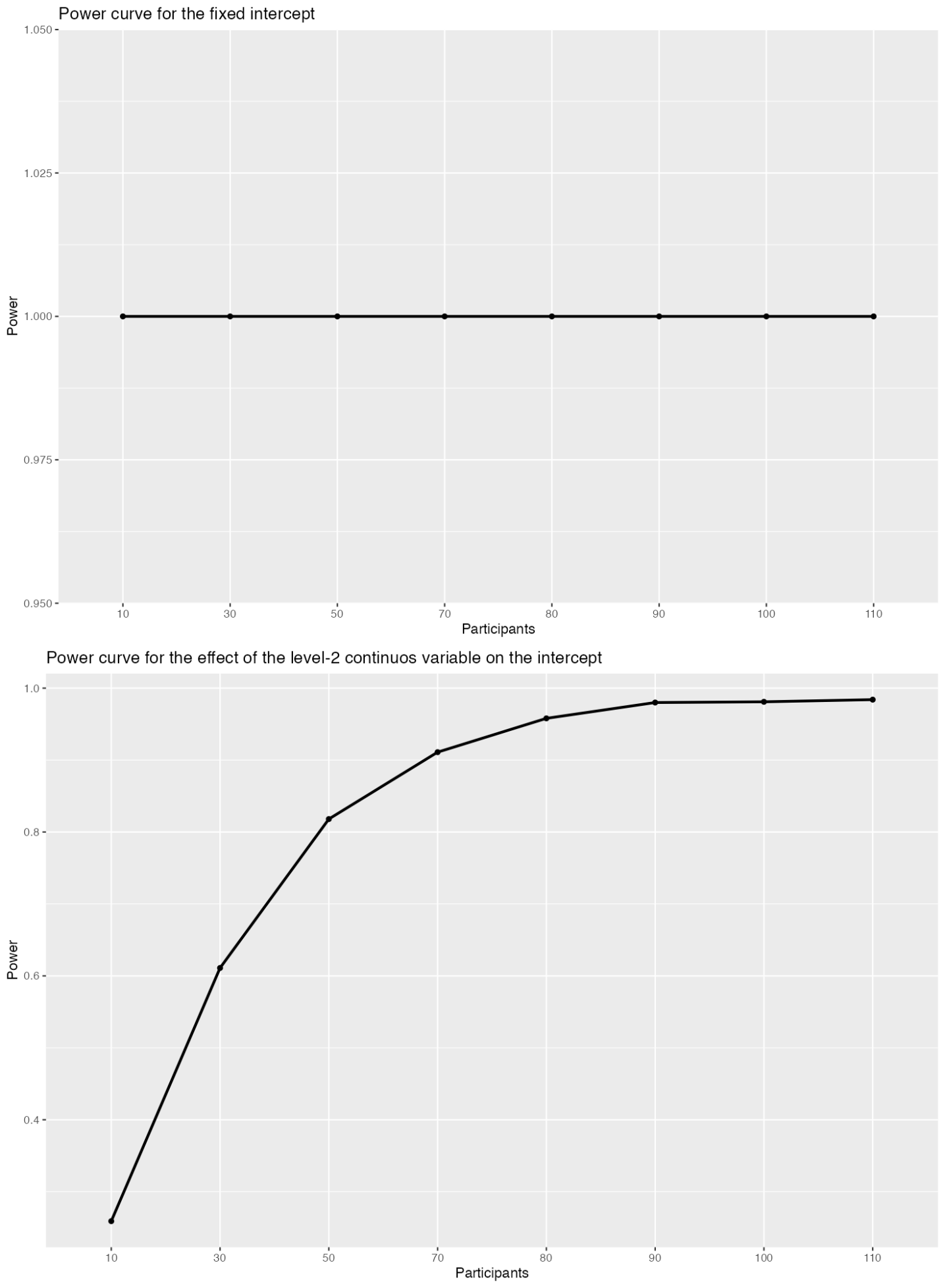


**Table S2. App User Experience.**

At the end of the study, 87% of responders reported that it was a normal week and that their mood, activities and contact with other people was not affected by using the app. Participants reported very few mistakes or technical issues. There were no group differences on any of the app user measures (Table S3).

| **Questions** | **Options** | **Whole Sample (N=68)** | **C (N=18)** | **MD (N=15)** | **HD (N=35)** | **Statistic** | **p** |  |
| --- | --- | --- | --- | --- | --- | --- | --- | --- |
| Do you feel this has been a normal week for you? | Yes/No | 59/9 | 15/3 | 14/1 | 30/5 | *Fisher’s Exact* | .803 |  |
| Did the participation in the PsyMate study affect your mood? | 1=worsened, 4=no affect, 7=increased | 3.75 (0.74) | 3.67 (0.59) | 3.67 (0.72) | 3.83 (0.82) | H(2, 68) = 0.62 | .734 |  |
| Did the participation in the PsyMate study affect your activities? | 1=decreased, 4=no affect, 7=increased | 4.1 (0.63) | 4.17 (0.62) | 4.13 (0.35) | 4.06 (0.73) | H(2, 68) = 1.43 | .489 |  |
| Did the participation in the PsyMate study affect your contacts with other people? | 1=decreased, 4=no affect, 7=increased | 3.9 (0.49) | 4.06 (0.54) | 3.87 (0.52) | 3.83 (0.45) | H(2, 68) = 2.14 | .343 |  |
| Did you make any mistakes answering the questions? | 1=none, 4=few, 7=many | 2.66 (1.25) | 2.44 (1.54) | 2.73 (1.22) | 2.74 (1.12) | H(2, 68) = 1.55 | .461 |  |
| Did you have technical difficulties using the PsyMate? | 1=not at all, 4=moderately, 7=very much | 2.47 (1.97) | 2.25 (1.34) | 3 (2) | 1.97 (1.52) | H(2, 68) = 3.56 | .169 |  |
| Mean (SD). Response rate was 68/80 (85%). Kruskal-Wallis test comparing between groups unless stated otherwise. | | | | | | | | |

**Table S3. Covariates Mood (PA and NA) and Assessment time predict Anticipation, Motivation and Enjoyment.**

| **Table S3** |  | **PA** | | | **NA** | | | | **Assessment time** | | |
| --- | --- | --- | --- | --- | --- | --- | --- | --- | --- | --- | --- |
|  | **Sub-processes** | **β** | **95% CI** | **p** | **β** | **95% CI** | **p** | **β** | | **95% CI** | **p** |
| **Activity** | Anticipation | 0.11 | 0.1 to 0.12 | <.001 | -0.06 | -0.08 to -0.05 | <.001 | 0.10 | | 0.08 to 0.12 | <.001 |
|  | Expectation | 0.49 | 0.3 to 0.68 | <.001 | -0.38 | -0.53 to -0.23 | <.001 | -0.03 | | -0.4 to 0.34 | .90 |
|  | Motivation (Interest) | 0.09 | 0.08 to 0.11 | <.001 | -0.05 | -0.06 to -0.04 | <.001 | 0.07 | | 0.03 to 0.11 | <.001 |
|  | Motivation (Prefer) | 0.12 | 0.1 to 0.14 | <.001 | -0.07 | -0.09 to -0.06 | <.001 | 0.12 | | 0.08 to 0.16 | <.001 |
|  | Enjoyment | 0.19 | 0.18 to 0.2 | <.001 | -0.09 | -0.1 to -0.08 | <.001 | 0.05 | | 0.01 to 0.09 | <.001 |
| **Social** | Anticipation | 0.09 | 0.08 to 0.11 | <.001 | -0.07 | -0.08 to -0.06 | <.001 | 0.06 | | 0.04 to 0.08 | <.001 |
|  | Enjoyment | 0.13 | 0.11 to 0.14 | <.001 | -0.09 | -0.1 to -0.08 | <.001 | 0.03 | | 0.01 to 0.05 | .02 |

**Table S4. Ranking of Activity by Enjoyment.**

| **Rank** | **Mean Enjoyment (SD)** | **Activity** | **Assessments** |
| --- | --- | --- | --- |
| 1 | 5.42 (1.41) | Exercising | 45 |
| 2 | 5.25 (1.35) | Relaxing | 634 |
| 3 | 5.22 (1.38) | Other leisure activity | 324 |
| 4 | 5.11 (1.43) | Eating/drinking | 284 |
| 5 | 4.93 (1.09) | Shopping | 58 |
| 6 | 4.46 (1.27) | Hygiene | 67 |
| 7 | 4.43 (1.42) | Social media | 121 |
| 8 | 4.06 (1.39) | Travelling | 153 |
| 9 | 3.77 (1.23) | Chores | 94 |
| 10 | 3.55 (1.58) | Nothing | 92 |
| 11 | 3.52 (1.49) | Studying | 176 |
| 12 | 3.46 (1.49) | Work/school | 251 |

**Table S5. Ranking of Company by Enjoyment.**

| **Rank** | **Mean Enjoyment (SD)** | **Company** | **Assessments** |
| --- | --- | --- | --- |
| 1 | 6.17 (1.42) | Partner | 236 |
| 2 | 5.82 (1.16) | Friends | 450 |
| 3 | 5.44 (1.37) | Family | 308 |
| 4 | 4.88 (1.57) | Nobody | 1,123 |
| 5 | 4.74 (1.05) | Colleague | 62 |
| 6 | 4.26 (1.15) | Acquaintances | 69 |
| 7 | 3.51 (1.32) | Strangers | 49 |

| **Table S6** | |  |  | | |
| --- | --- | --- | --- | --- | --- |
|  | **Sub-processes** | | **β** | **95% CI** | **p** |
| **Activity** | Anticipatory Pleasure | | -0.019 | -0.033 to -0.005 | 0.008 |
|  | Expectation | | -0.265 | -0.502 to -0.028 | 0.031 |
|  | Motivation (Interest) | | -0.015 | -0.028 to -0.002 | 0.026 |
|  | Motivation (Prefer) | | -0.019 | -0.033 to -0.005 | 0.010 |
|  | Enjoyment | | -0.016 | -0.028 to -0.004 | 0.009 |
| Multilevel linear models with MFQ as predictor. Controlled for assessment time, and mood (NA and PA). | | | | | |

**Table S6. Depression as a predictor of activity anticipation, motivation and enjoyment.**

**Table S7. Depression predicting anticipation, motivation and enjoyment of leisure and functional activity.**

| **Table S7** | | | | | | | |  |  |
| --- | --- | --- | --- | --- | --- | --- | --- | --- | --- |
|  | **Leisure** | | | **Functional** | | | | |  |
| **Sub-processes** | **β** | **95% CI** | **p** | | **β** | **95% CI** | **p** | | |
| Anticipatory Pleasure | -0.013 | -0.026 to 0.001 | 0.076 | | -0.014 | -0.029 to 0.001 | 0.068 | | |
| Expectation | -0.288 | -0.543 to -0.034 | 0.029 | | -0.245 | -0.484 to -0.006 | 0.048 | | |
| Motivation (Interest) | -0.013 | -0.027 to 0.001 | 0.071 | | -0.009 | -0.024 to 0.005 | 0.203 | | |
| Motivation  (Prefer) | -0.014 | -0.029 to 0.001 | 0.070 | | -0.012 | -0.029 to 0.005 | 0.175 | | |
| Enjoyment | -0.019 | -0.033 to -0.005 | 0.011 | | -0.012 | -0.024 to 0 | 0.048 | | |
| Multilevel linear models with MFQ as predictor. Controlled for assessment time, and mood (NA and PA). Leisure (exercise, relaxing and other leisure) functional (work/school, studying, chores, shopping, hygiene, eating/drinking, travelling). | | | | | | | |  |  |

**Table S8. Depression as a predictor of company enjoyment.**

| **Table S8** | |  |  | | |
| --- | --- | --- | --- | --- | --- |
|  | **Sub-processes** | | **β** | **95% CI** | **p** |
| **Company** | Anticipatory Pleasure | | -0.017 | -0.033 to -0.002 | 0.033 |
|  | Enjoyment | | -0.019 | -0.033 to -0.004 | 0.012 |
| Multilevel linear models with MFQ as predictor. Controlled for assessment time, and mood (NA and PA). | | | | | |

**Table S9. Depression as a predictor of anticipation and enjoyment of social and non-social company.**

| **Table S9** | | | | | | |  |  |
| --- | --- | --- | --- | --- | --- | --- | --- | --- |
|  | **Social** | | | **Non-Social** | | | | |
| **Sub-processes** | **β** | **95% CI** | **p** | **β** | **95% CI** | **p** | |  |
| Anticipatory Pleasure | -0.027 | -0.042 to -0.012 | 0.001 | -0.017 | -0.037 to 0.004 | 0.120 | |  |
| Enjoyment | -0.020 | -0.036 to -0.005 | 0.013 | -0.023 | -0.043 to -0.004 | 0.023 | |  |
| Multilevel linear models with MFQ as predictor. Controlled for assessment time, and mood (NA and PA). Social (Friends, family, partner) Non-social (nobody). | | | | | | |  |  |

**Table S10. Depression as a predictor of specific activity and company enjoyment.**

| **Table S10** |  |  |  |  |
| --- | --- | --- | --- | --- |
|  |  | **β** | **95% CI** | **p** |
| **Activity** | Relaxing | -0.010 | -0.026 to 0.005 | .207 |
|  | Work/school | -0.014 | -0.032 to 0.005 | .159 |
|  | Studying | -0.032 | -0.05 to -0.013 | .005 |
|  | Chores | -0.019 | -0.046 to 0.006 | .164 |
|  | Shopping | 0.003 | -0.021 to 0.026 | .831 |
|  | Hygiene | 0.009 | -0.02 to 0.039 | .534 |
|  | Eating/drinking | -0.022 | -0.04 to -0.004 | .022 |
|  | Travelling | -0.018 | -0.041 to 0.005 | .130 |
|  | Social media | -0.021 | -0.05 to 0.007 | .152 |
|  | Exercising | -0.003 | -0.044 to 0.04 | .907 |
|  | Other leisure activity | -0.020 | -0.036 to -0.004 | .018 |
|  | Nothing | -0.018 | -0.053 to 0.018 | .330 |
| **Company** | Partner | -0.009 | -0.039 to 0.02 | .537 |
|  | Friends | -0.013 | -0.033 to 0.007 | .207 |
|  | Family | -0.018 | -0.044 to 0.008 | .189 |
|  | Colleague | 0.003 | -0.019 to 0.026 | .774 |
|  | Acquaintances | 0.003 | -0.025 to 0.031 | .825 |
|  | Strangers | 0.026 | -0.021 to 0.075 | .293 |
|  | Nobody | -0.017 | -0.037 to 0.003 | .101 |
| Multilevel linear models with MFQ as a predictor of Enjoyment. Controlled for assessment time, and mood (NA and PA). | | | | |

| **Table S11** |  |  |  |  |
| --- | --- | --- | --- | --- |
|  |  | **β** | **95% CI** | **p** |
| **Activity** | Relaxing | -0.084 | -0.333 to 0.165 | 0.511 |
|  | Work/school | -0.256 | -0.458 to -0.054 | 0.016 |
|  | Studying | -0.180 | -0.39 to 0.03 | 0.098 |
|  | Chores | -0.065 | -0.243 to 0.113 | 0.478 |
|  | Shopping | 0.030 | -0.035 to 0.095 | 0.366 |
|  | Hygiene | -0.088 | -0.166 to -0.01 | 0.033 |
|  | Eating/drinking | 0.078 | -0.051 to 0.207 | 0.241 |
|  | Travelling | 0.003 | -0.115 to 0.121 | 0.954 |
|  | Social media | 0.054 | -0.095 to 0.203 | 0.481 |
|  | Exercising | -0.041 | -0.127 to 0.045 | 0.356 |
|  | Other leisure activity | -0.033 | -0.227 to 0.161 | 0.740 |
|  | Nothing | 0.170 | 0.027 to 0.313 | 0.026 |
| **Company** | Partner | -0.341 | -0.721 to 0.039 | 0.086 |
|  | Friends | 0.002 | -0.357 to 0.361 | 0.993 |
|  | Family | 0.053 | -0.296 to 0.402 | 0.766 |
|  | Colleague | -0.165 | -0.465 to 0.135 | 0.294 |
|  | Acquaintances | 0.204 | -0.108 to 0.516 | 0.208 |
|  | Strangers | -0.031 | -0.315 to 0.253 | 0.831 |
|  | Nobody | -0.067 | -0.414 to 0.28 | 0.707 |
| Multilevel linear models with MFQ as a predictor of Engagement. Controlled for assessment time, and mood (NA and PA). Engagement was calculated as the % of a subject’s total assessments spent in each activity. | | | | |

**Table S11. Depression as a predictor of specific activity and company engagement.**

**Time-lagged analyses:**

**Table S12. Reward predictors of activity enjoyment and company enjoyment, when predictors and outcomes are for the same events.**

| **Table 12a Activity Enjoyment** | | | | | | | | | | | | | | | | |
| --- | --- | --- | --- | --- | --- | --- | --- | --- | --- | --- | --- | --- | --- | --- | --- | --- |
|  | **Whole Sample (n = 106)** | | | | **C (n = 22)** | | | | **MD (n = 19)** | | | | **HD (n = 65)** | | | |
| **Predictors (t-1)** | **β** | **95% CI** | **p** | **β** | | **95% CI** | **p** | **β** | | **95% CI** | **p** | **β** | | **95% CI** | **p** |  |
| (Intercept) | 4.733 | 3.808 to 5.658 | <.001 | 5.694 | | 3.132 to 8.256 | .001 | 5.222 | | 2.684 to 7.76 | .003 | 4.231 | | 3.041 to 5.421 | <.001 |  |
| Anticipatory Pleasure | 0.648 | 0.44 to 0.856 | <.001 | 0.134 | | -0.528 to 0.796 | .697 | 0.741 | | -0.072 to 1.554 | .112 | 0.723 | | 0.476 to 0.97 | <.001 |  |
| Expectation | -0.007 | -0.023 to 0.009 | .376 | 0.010 | | -0.041 to 0.061 | .704 | 0.004 | | -0.047 to 0.055 | .884 | -0.012 | | -0.03 to 0.006 | 0.182 |  |
| **Table 12b Company Enjoyment** | | | | | | | | | | | | | | | | |
|  | **Whole Sample (n = 412)** | | | | **C (n = 102)** | | | | **MD (n = 51)** | | | | **HD (n = 259)** | | | |
| **Predictors (t-1)** | **β** | **95% CI** | **p** | **β** | | **95% CI** | **p** | **β** | | **95% CI** | **p** | **β** | | **95% CI** | **p** |  |
| (Intercept) | 5.241 | 4.996 to 5.486 | <.001 | 5.555 | | 5.094 to 6.016 | <.001 | 5.588 | | 5.086 to 6.09 | <.001 | 4.971 | | 4.64 to 5.302 | <.001 |  |
| Anticipatory Pleasure | 0.660 | 0.562 to 0.758 | <.001 | 0.686 | | 0.474 to 0.898 | <.001 | 0.603 | | 0.344 to 0.862 | <.001 | 0.661 | | 0.538 to 0.784 | <.001 |  |
| Time-lagged linear regressions, predictors (t-1) and enjoyment (t) are matched for the same events e.g., relaxing to relaxing, friends to friends. Controlled for assessment time, and mood (NA and PA). When age and assessment period added as extra covariates, results remain the same. | | | | | | | | | | | | | | | | |

**Table S13. Reward predictors of leisure enjoyment and social company enjoyment.**

| **Table S13a Leisure activity enjoyment** | | | | | | | | | | | | | | | | |  |
| --- | --- | --- | --- | --- | --- | --- | --- | --- | --- | --- | --- | --- | --- | --- | --- | --- | --- |
|  | **Whole Sample (n = 325)** | | | | **C (n = 111)** | | | | **MD (n = 51)** | | | | **HD (n = 163)** | | | |  |
| **Predictors (t-1)** | **β** | **95% CI** | **p** | **β** | | **95% CI** | **p** | **β** | | **95% CI** | **p** | **β** | | **95% CI** | **p** |  |  |
| (Intercept) | 5.020 | 4.634 to 5.406 | <.001 | 5.277 | | 4.652 to 5.902 | <.001 | 4.866 | | 3.835 to 5.897 | <.001 | 4.642 | | 4.054 to 5.23 | <.001 |  |  |
| Anticipatory Pleasure | -0.060 | -0.203 to 0.083 | .408 | 0.008 | | -0.251 to 0.267 | .949 | -0.013 | | -0.478 to 0.452 | .957 | -0.071 | | -0.269 to 0.127 | .483 |  |  |
| Expectation | 0.003 | -0.005 to 0.011 | .462 | -0.007 | | -0.023 to 0.009 | .354 | -0.002 | | -0.02 to 0.016 | .797 | 0.007 | | -0.005 to 0.019 | .201 |  |  |
| Motivation (Interest) | 0.127 | -0.006 to 0.26 | .062 | 0.114 | | -0.123 to 0.351 | .347 | 0.256 | | -0.069 to 0.581 | .130 | 0.094 | | -0.1 to 0.288 | .346 |  |  |
| Motivation (Prefer) | 0.047 | -0.043 to 0.137 | .308 | 0.047 | | -0.1 to 0.194 | .532 | 0.003 | | -0.273 to 0.279 | .982 | 0.045 | | -0.082 to 0.172 | .492 |  |  |
| **Table S13b Social company enjoyment** | | | | | | | | | | | | | | | |  |  |
|  | **Whole Sample (n = 694)** | | | **C (n = 179)** | | | | **MD (n = 133)** | | | | **HD (n = 382)** | | | |  | |
| **Predictors (t-1)** | **β** | **95% CI** | **p** | **β** | | **95% CI** | **p** | **β** | | **95% CI** | **p** | **β** | | **95% CI** | **p** |  |  |
| (Intercept) | 5.735 | 5.529 to 5.941 | <.001 | 6.186 | | 5.963 to 6.409 | <.001 | 5.860 | | 5.437 to 6.283 | <.001 | 5.471 | | 5.165 to 5.777 | <.001 |  |  |
| Anticipatory Pleasure | 0.188 | 0.127 to 0.249 | <.001 | 0.076 | | -0.036 to 0.188 | .181 | 0.155 | | -0.002 to 0.312 | .055 | 0.223 | | 0.145 to 0.301 | <.001 |  |  |
| Time-lagged linear regressions. Controlled for assessment time, and mood (NA and PA). When age and assessment period added as extra covariates, results remain the same. | | | | | | | | | | | | | | | | |  |

**Table S14. Reward predictors of leisure engagement and social company engagement, when predictors and outcomes are for the same events.**

| **Table S14a Leisure activity engagement** | | | | | | | | | | | | | | | | |
| --- | --- | --- | --- | --- | --- | --- | --- | --- | --- | --- | --- | --- | --- | --- | --- | --- |
|  | **Whole Sample (n = 408)** | | | | **C (n = 122)** | | | | **MD (n = 70)** | | | | **HD (n = 216)** | | | |
| **Predictors (t-1)** | **β** | **95% CI** | **p** | **β** | | **95% CI** | **p** | **β** | | **95% CI** | **p** | **β** | | **95% CI** | **p** |  |
| (Intercept) | 5.509 | 5.135 to 5.883 | <.001 | 6.163 | | 5.681 to 6.645 | <.001 | 5.437 | | 4.496 to 6.378 | <.001 | 5.183 | | 4.613 to 5.753 | <.001 |  |
| Anticipatory Pleasure | 0.182 | 0.088 to 0.276 | <.001 | 0.034 | | -0.142 to 0.21 | .703 | 0.137 | | -0.11 to 0.384 | .284 | 0.222 | | 0.091 to 0.353 | .001 |  |
| **Table S14b Social company engagement** | | | | | | | | | | | | | | | | |
|  | **Whole Sample (n = 396)** | | | | **C (n = 97)** | | | | **MD (n = 51)** | | | | **HD (n = 248)** | | | |
| **Predictors (t-1)** | **β** | **95% CI** | **p** | **β** | | **95% CI** | **p** | **β** | | **95% CI** | **p** | **β** | | **95% CI** | **p** |  |
| (Intercept) | -0.512 | -1.049 to 0.025 | .061 | -0.722 | | -2.127 to 0.683 | .313 | -0.641 | | -1.505 to 0.223 | .146 | -0.393 | | -1.112 to 0.326 | .284 |  |
| Anticipatory Pleasure | 0.254 | 0.021 to 0.487 | .033 | 0.855 | | 0.151 to 1.559 | .017 | 0.439 | | -0.394 to 1.272 | .301 | 0.114 | | -0.149 to 0.377 | .396 |  |
| Time-lagged linear regressions, predictors (t-1) and engagement (t) are matched for the same events e.g., relaxing to relaxing, friends to friends. Controlled for assessment time, and mood (NA and PA). When age and assessment period added as extra covariates, results remain the same. | | | | | | | | | | | | | | | | |

**References**

1. Kring, A.M. and D.M. Barch, *The motivation and pleasure dimension of negative symptoms: Neural substrates and behavioral outputs.* European Neuropsychopharmacology, 2014. **24**(5): p. 725-736.

2. Lafit, G., et al., *Selection of the Number of Participants in Intensive Longitudinal Studies: A User-Friendly Shiny App and Tutorial for Performing Power Analysis in Multilevel Regression Models That Account for Temporal Dependencies.* Advances in Methods and Practices in Psychological Science, 2021. **4**(1): p. 2515245920978738.

3. Li, X., et al., *Diminished Anticipatory and Consummatory Pleasure in Dysphoria: Evidence From an Experience Sampling Study.* Frontiers in psychology, 2019. **10**.

4. van Roekel, E., L. Keijsers, and J.M. Chung, *A review of current ambulatory assessment studies in adolescent samples and practical recommendations.* Journal of Research on Adolescence, 2019. **29**(3): p. 560-577.

5. Eisele, G., et al., *The effects of sampling frequency and questionnaire length on perceived burden, compliance, and careless responding in experience sampling data in a student population.* Assessment, 2022. **29**(2): p. 136-151.
