# Supplementary figures and images for: Anticipation and Motivation as Predictors of Leisure and Social Enjoyment and Engagement in Young People With Depression Symptoms: Ecological Momentary Assessment Study
Source: JMIR Ment Health. 2025 Aug 13;12:e74427. doi: 10.2196/74427 (PMC12349889; doi:10.2196/74427)

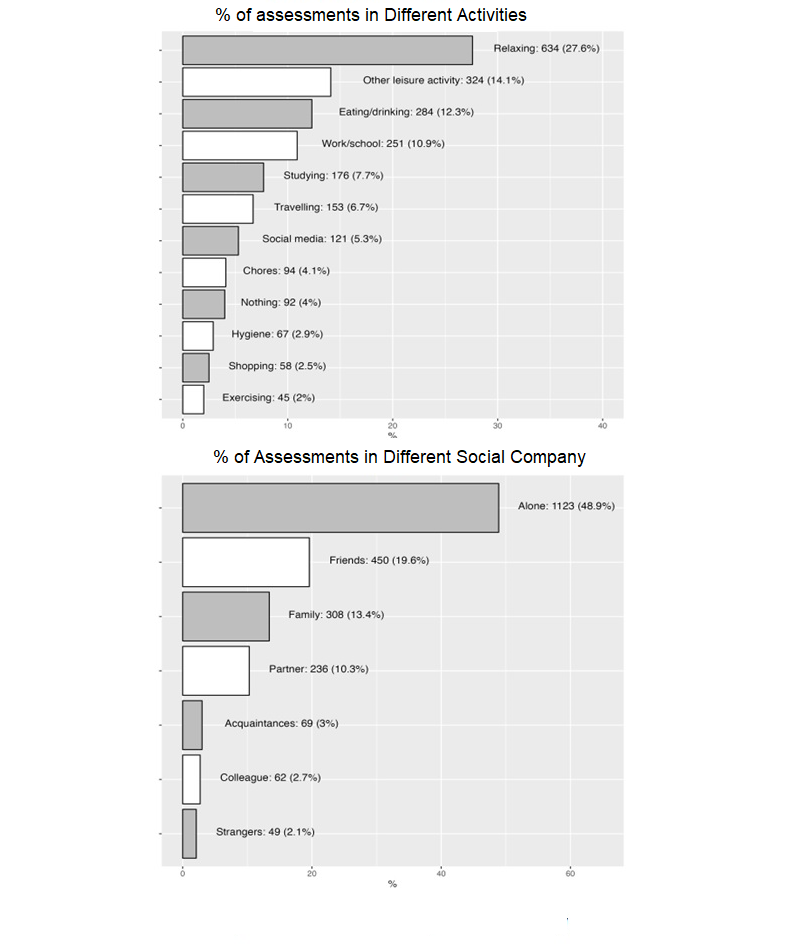

Supplement: Multimedia Appendix 2 [file mental-v12-e74427-s002.png]
